# Estrogen receptor β induces autophagy of osteosarcoma through the mTOR signaling pathway

**DOI:** 10.1186/s13018-020-1575-1

**Published:** 2020-02-13

**Authors:** Zhengming Yang, Wei Yu, Bing Liu, Minfei Yang, Huimin Tao

**Affiliations:** 1 Department of Orthopaedics, Second Affiliated Hospital, School of Medicine, Zhejiang University, No.1511 Jianghong Road, Binjiang District, Zhejiang, 310000 Hangzhou China; 2 Department of Emergency Room, Second Affiliated Hospital, School of Medicine, Zhejiang University, Zhejiang, 310000 Hangzhou China

**Keywords:** Osteosarcoma, Estrogen receptor beta, Autophagy, mTOR

## Abstract

**Background:**

Estrogen receptor beta (ERβ) was considered as a tumor-inhibiting factor in estrogen-sensitive malignant tumors. In this study, we intended to investigate whether ERβ was involved in inducing autophagy in osteosarcoma.

**Methods:**

This is an experimental study. The associations between ERβ and autophagy were detected in osteosarcoma U2-OS cells which were treated with E2, E2 + 2,3-Bis (4-hydroxyphenyl) propionitrile (DPN, ERβ agonists), E2 + DPN + water, E2 + DPN + 3-Methyladenine (3-MA, autophagy inhibitor), respectively. Cell viability and death were detected using cell counting kit 8 assay and flow cytometry, respectively. In addition, the expression of autophagy marker LC3II/I, sequestosome 1 (P62), mammalian target of rapamycin (mTOR), and phosphorylated-mTOR (p-mTOR) was determined by reverse transcription quantitative polymerase chain reaction (RT-qPCR) and Western blotting.

**Results:**

Cell viability was significantly decreased with DPN treatment, while was reversed with 3-MA treatment. DPN treatment decreased living cells proportion and increased cell apoptosis proportion, while 3-MA treatment reversed those changes. However, there were significant differences between the E2 group and the E2 + DPN + 3-MA group for the living cell proportion and cell apoptosis proportion, suggesting apoptosis and autophagy all were induced. In addition, DPN treatment upregulated the LC3II/I expression level and downregulated P62 and mTOR (mRNA level) and p-mTOR (protein level) expression levels.

**Conclusion:**

ERβ inhibited the cell viability and mediated cell death by inducing apoptosis and autophagy in osteosarcoma. ERβ-induced autophagy in osteosarcoma was associated with downregulating the P62 expression level and inhibiting mTOR activation.

## Background

Osteosarcoma is the most common primary malignant tumor in bone tissue that mainly occurs in adolescents and the elderly, and it is characterized by proliferating tumor cells that directly form bone or bone-like tissue [1]. The incidence of osteosarcoma in males is higher than that in females and commonly emerges in the distal femur, proximal tibia, and proximal humerus [2]. The current treatment of osteosarcoma is mainly neoadjuvant chemotherapy and surgery, and the 5-year overall survival rate has increased to about 70 to 80% because of the introduction of neoadjuvant chemotherapy and radiotherapy [3, 4]. However, the prognosis of patients with osteosarcoma is poor, and metastasis is the most important prognostic factor, followed by chemotherapy response, tumor characteristics, relapse, and so on [4]. The overall survival of the patients with metastasis or recurrence is less than 20% in spite of chemotherapeutic drugs [5]. In addition, drug-resistance and severe side effects, including cardiotoxicity and nephrotoxicity, are the adverse factors of chemotherapy [6]. Therefore, it is necessary to further study the inner mechanism of the development and progression of osteosarcoma to provide a theoretical basis for clinical treatment.

Estrogen receptors (ER) belong to the nuclear receptor superfamily, and these ligand-dependent receptors respond to the presence of estrogen [7]. The changes in the ER expression level or tissue distribution are observed in multiple cancers [8]. The two subtypes, ERα and ERβ, are highly homologous in structure, while being different in tissue distribution and the biological effects under binding to ligand [9]. Reportedly, ERβ serves an important role in autophagy. For instance, Pierdominici et al. reported that ERβ activation can repress the progression of Hodgkin lymphoma by inducing autophagy [10]. Nevertheless, there are no reports focusing on whether ERβ is implicated in mediating autophagy in osteosarcoma. In our previous study, we found that ERβ mediated the proliferation, migration, and invasion of osteosarcoma cells by the regulation of the integrin, Bcl-2, and PI3K/Akt signal pathways [11]. Hence, we intended to investigate whether ERβ is involved in inducing autophagy in osteosarcoma. 2,3-Bis (4-hydroxyphenyl) propionitrile (DPN) was a selective agonist of ERβ and it had been used to examine the role of ERβ in studies [12, 13], while 3-methyladenine (3-MA) was an inhibitor of autophagy [14]. In our study, DPN and 3-MA were selected to examine the association among ERβ and autophagy on osteosarcoma cell lines U2-OS.

## Methods

### Cell culture and treatment

The osteosarcoma cell lines U2-OS were purchased from the Cell Bank of the Chinese Academy of Sciences (Shanghai, China), and cultured in RPMI-1640 medium (catalog no. C22400500BT, Gibco) supplemented with 10% fetal bovine serum (FBS, catalog no. 10099-141, Gibco) and 1% penicillin-streptomycin (catalog no. BS734, Sangon Biotech) in an incubator with 5% CO_2_ at 37 °C. The U2-OS cells under logarithmic growth phase were used in subsequent experiments. There were four groups in this study.

- Group A: NC + 1 uM E2 (estradiol; catalog no. E808987, Macklin);
- Group B: NC + 1 uM E2 + 100 uM DPN (catalog no. H5915, Sigma);
- Group C: NC + 1 uM E2 + 100 uM DPN + water (solvent);
- Group D: NC + 1 uM E2 + 100uM DPN + 1 mM 3-MA (catalog no. HY-19312, MCE).

The concentrations of E2, DPN, and 3-MA were determined by half-maximal inhibitory concentration (IC50).

### Cell counting kit-8 assay

Cells suspension was prepared at 5 × 10^4^ cells/ml and placed at 100 μl/well in 96-well plate overnight at 37 °C. After 48 h of treatments, the original medium was replaced with serum-free medium containing 10% of 5 mg/ml cell counting kit-8 (CCK-8) solution (catalog no. C0039, Beyotime) at 100 μl/well in 96-well plate, and incubated in dark for 2 h. Then, the light absorption value of each well at 450 nm was measured using a microplate reader (Infinite M100 PRO, TECAN).

### Apoptosis analysis

Cells were placed in a six-well plate at a density of 3 × 10^5^ cells/well overnight, and three duplicates were set for each group. After 48 h of treatments, apoptosis was determined by flow cytometric analysis. In short, cells were resuspended in 100 μL 1× binding buffer coupled with 5 μl FITC-Annexin-V (catalog no. 40301ES50, BD) and 5 μl of 50 μg/mL propidium iodide (Sigma), and incubated in the dark for 2 h at room temperature. Then, cell apoptosis was measured using FACS Calibur flow cytometer (BD) after adding 400 μL of 1x binding buffer.

### Reverse transcription quantitative polymerase chain reaction

Total RNA was extracted using TRIzol reagent (catalog no. 15596026, Invitrogen; Thermo) in the light of instructions. The quality and purity of total RNA were measured by a microplate reader (Infinite M100 PRO, TECAN). The total RNA was reverse transcribed using PrimeScript™ RT Master Mix (catalog no. RR036A, Takara). Then, RT-qPCR was performed using Power SYBR Green PCR Master Mix (catalog no. A25742, Thermo). The thermal cycling conditions were as follows: 50 °C for 3 min, 95 °C for 3 min, followed by 40 cycles at 95 °C for 10 s and 60 °C for 30 s. Melt curve was analyzed from 60 to 95 °C at an increment rate of 0.5 °C/10 s. The relative expression of the genes was calculated by the 2^−ΔΔCt^ method [15]. The primer sequences for the genes were as follows: microtubule-associated protein 1 Light Chain 3 (LC3), LC3II/I, forward 5′-AACATGAGCGAGTTGGTCAAG-3′; LC3II/I, reverse 5′-GCTCGTAGATGTCCGCGAT-3′; Sequestosome 1 (P62), forward 5′-GGAACAGCGACTCTTGCTTC-3′; reverse 5′-GGTGCTCGATATGGCATTAGTG-3′; mammalian target of rapamycin (mTOR), forward 5′-TCCGAGAGATGAGTCAAGAGG-3′; reverse 5′-CACCTTCCACTCCTATGAGGC-3′;GAPDH, forward 5′-TGACAACTTTGGTATCGTGGAAGG-3′; GAPDH, reverse 5′-AGGCAGGGATGATGTTCTGGAGAG-3′.

### Western blotting

Cells were lysed in RIPA lysis buffer (catalog no. P0013B, Beyotime) coupled with PMSF (catalog no. ST506, Beyotime) at a final concentration of 1 mM, and the protein concentration was determined using a BCA Protein Assay Kit (catalog no. 23227, Thermo). Protein (20 μg) from each sample was separated by 10% SDS-polyacrylamide gel and transferred onto polyvinylidene difluoride (PVDF) membranes (catalog no. IPVH00010, Millipore). Five percent of skim milk (catalog no. 232100, BD) was used to block the nonspecific binding for 1–2 h at 37 °C and then incubated with anti-LC3I/II (1:1000, catalog no. WL01506, Wanleibio), anti-p62 (1:1000, catalog no. 5114S, CST), anti-p65 (1:1000, catalog no. 1074S, CST), anti-P mTOR (1:1000, catalog no. 5536S, CST), anti-mTOR (1:1000, catalog no. 20657-1-AP, Proteintech), anti-β actin (1:1000, catalog no. ab8226, abcam) primary antibody at 4 °C overnight. The membrane was washed with PBST buffer and then incubated with HRP secondary antibodies (1:10000, catalog no.111-035-045, Jackson) for 2 h at 37 °C. After washing with PBST buffer six times, the membrane was exposed with enhanced chemiluminescence (ECL) reagents (catalog no. SB-WB012, Share-bio) and the Millipore ECL system, and then gray scanning was performed using TanonImage (4600, Tanon).

### Autophagic flux assay

Cells were placed in a six-well plate at a density of 2 × 10^5^ cells/well, and pLV-GFP-LC3 lentivirus with multiplicity of infection (MOI) of150 and 6 μg/ml of polybrene (catalog no. 107689, sigma) were added. Then, cells were transferred to a 10-cm dish, and puromycin (catalog no. P8833, Ssigma) was added with a final concentration of 2 μg/ml for 72 h when the cell density reached 60%. Finally, the cells transfected with pLV-GFP-LC3 lentivirus were treated with different drugs. The expression of green fluorescence was detected using fluorescence microscopy (TCS SP5, Leica) after 48-h treatments.

### Statistical analysis

Statistical analysis was performed using SPSS 22.0 software package (SPSS Inc., Chicago, IL). All experiments were performed in triplicate, and the results were presented with mean value ± standard deviation (SD). The Shapiro-Wilk was used to test the normality of the distribution. For the data presenting a normal distribution, Student’s *t* test (two groups) and one-way ANOVA (more than two groups) were used to compare the results among different groups. The Wilcoxon rank-sum test was used for nonnormally distributed data. *P* < 0.05 was selected to show the significant difference.

## Results

### ERβ inhibited the viability of U2-OS cells

**Fig. 1:**
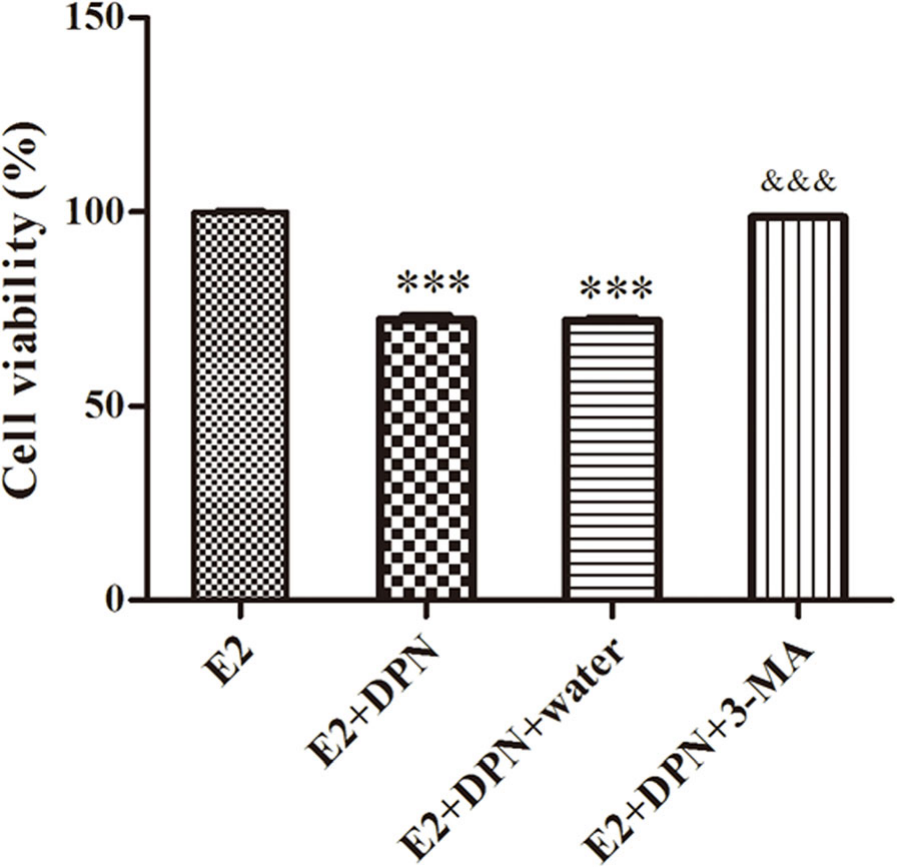
The U2-OS cells viability detected by CCK-8 assay. *: E2 vs. E2 + DPN; E2 vs. E2 + DPN + water; ^&^: E2 + DPN + 3-MA vs. E2 + DPN; E2 + DPN + 3-MA vs. E2 + DPN + water. *** *P* < 0.001; ^&&&^*P* < 0.001

The cells viability were inhibited with DPN treatment compared with that in control (E2 vs. E2 + DPN; E2 vs. E2 + DPN + water; *P* < 0.001), while the inhibited cells viability were reversed with 3-MA treatment (E2 + DPN + 3-MA vs. E2 + DPN; E2 + DPN + 3-MA vs. E2 + DPN + water; *P* < 0.001), indicating that ERβ could inhibit the osteosarcoma cells viability probably through inducing autophagy (Fig. 1).

### ERβ mediates U2-OS cell death by inducing apoptosis and autophagy

**Fig. 2:**
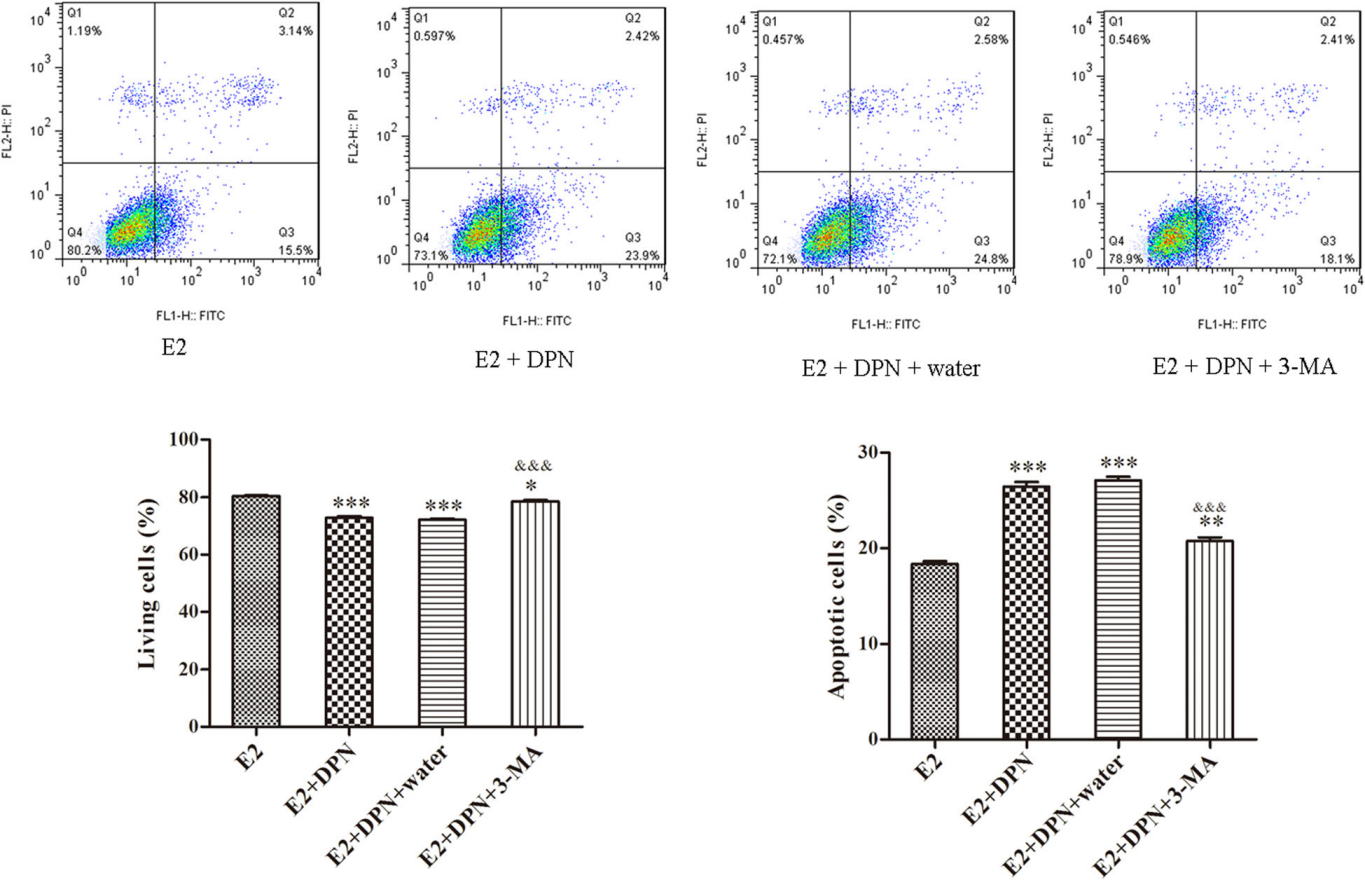
Survival and apoptosis of U2-OS cells detected by flow cytometry. *: E2 vs. E2 + DPN; E2 vs. E2 + DPN + water; E2 vs. E2 + DPN + 3-MA ^&^: E2 + DPN + 3-MA vs. E2 + DPN; E2 + DPN + 3-MA vs. E2 + DPN + water. *P < 0.05; *** *P* < 0.001; ^&&&^*P* < 0.001

The proportion of living cells were decreased with DPN treatment compared with the control (E2 vs. E2 + DPN; E2 vs. E2 + DPN + water; *P* < 0.001). Meanwhile, the proportion of living cells were also decreased with 3-MA treatment compared with control (E2 vs. E2 + DPN + 3-MA, *P* < 0.05), while it was increased compared with DPN treatment (E2 + DPN + 3-MA vs. E2 + DPN; E2 + DPN + 3-MA vs. E2 + DPN + water; *P* < 0.001). In addition, cell apoptosis was induced with DPN treatment compared with control (E2 vs. E2 + DPN; E2 vs. E2 + DPN + water; *P* < 0.001). The proportion of apoptotic cells were also increased with 3-MA treatment compared with control (E2 vs. E2 + DPN + 3-MA, *P* < 0.01), while it was decreased compared with DPN treatment (E2 + DPN + 3-MA vs. E2 + DPN; E2 + DPN + 3-MA vs. E2 + DPN + water; *P* < 0.001). Those findings suggested that ERβ could mediate cell death in osteosarcoma by inducing apoptosis and autophagy (Fig. 2).

**Fig. 3:**
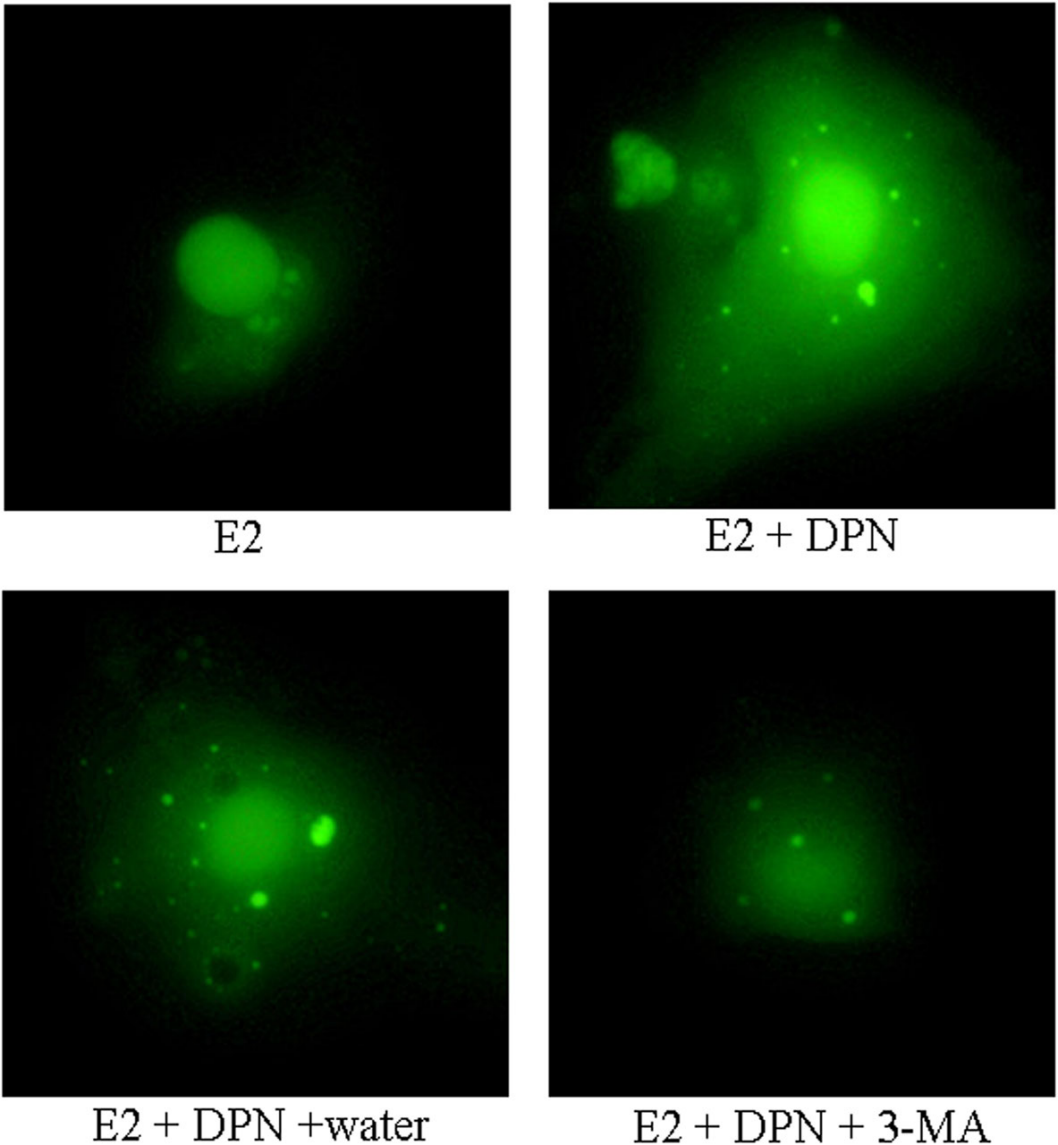
GFP-LC3 single fluorescence autophagy assay. Green fluorescence spots represent autophagosomes, more green fluorescence spots represent an increase of autophagy

Moreover, the GFP-LC3 fusion protein was dispersed in the cytoplasm in control (E2 group), while there were many green fluorescence spots with DPN treatment (E2 + DPN and E2 + DPN + water group), that were autophagosomes. However, autophagosomes were decreased in E2 + DPN + 3-MA group. The results of autophagic flux assay revealed that ERβ induced autophagy in osteosarcoma cells (Fig. 3).

### The expression of LC3-II/I, P62, and mTOR at mRNA and protein levels

**Fig. 4:**
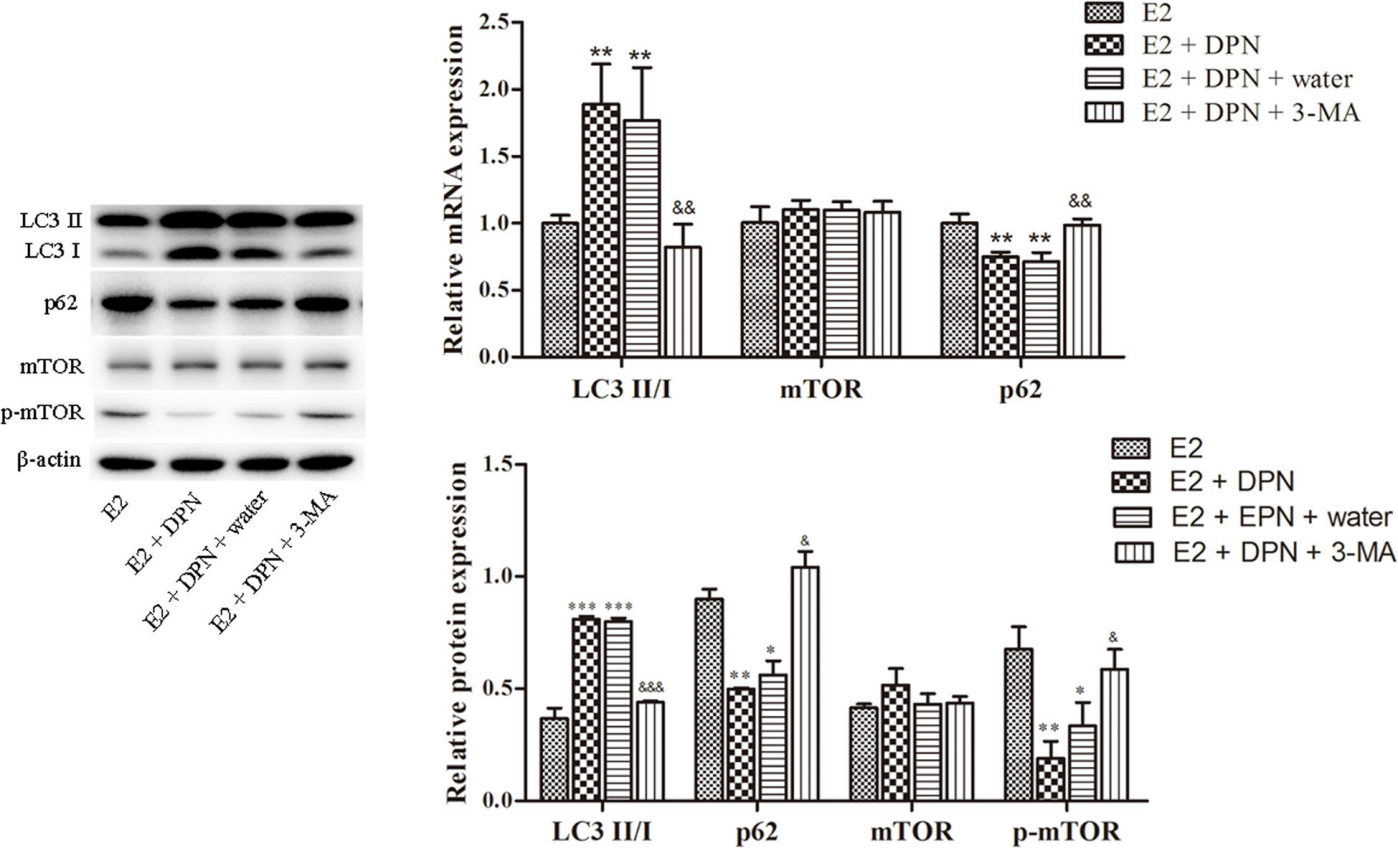
The expression of LC3-II/I, P62, mTOR, and p-mTOR determined by RT-qPCR and Western blotting. *: E2 vs. E2 + DPN; E2 vs. E2 + DPN + water; E2 vs. E2 + DPN + 3-MA. ^&^: E2 + DPN + 3-MA vs. E2 + DPN; E2 + DPN + 3-MA vs. E2 + DPN + water. * *P* < 0.05; ^&^ P < 0.05; ** *P* < 0.01; ^&&^*P* < 0.01; *** *P* < 0.001; ^&&&^*P* < 0.001

To demonstrate the mechanisms of ERβ-induced autophagy in U2-OS cells, the expression of LC3-II/I, P62, and mTOR were determined (Fig. 4). LC3 was an autophagy marker, and LC3-I conversion to LC3-II occurred during autophagy. The expression of LC3-II/I was increased with DPN treatment compared with control (E2 vs. E2 + DPN; E2 vs. E2 + DPN + water; *P* < 0.01 at mRNA level, *P* < 0.001 at protein level), while the increased LC3-II/I expression was reversed with 3-MA treatment (E2 + DPN + 3-MA vs. E2 + DPN; E2 + DPN + 3-MA vs. E2 + DPN + water; *P* < 0.01 at mRNA level, *P* < 0.001 at protein level).

Nevertheless, the mRNA expression of P62 was decreased with DPN treatment compared with control (E2 vs. E2 + DPN; E2 vs. E2 + DPN + water; *P* < 0.01), and was reversed with 3-MA treatment (E2 + DPN + 3-MA vs. E2 + DPN; E2 + DPN + 3-MA vs. E2 + DPN + water; *P* < 0.01). Also, the protein expression of P62 was decreased with DPN treatment compared with control (E2 vs. E2 + DPN, *P* < 0.01; E2 vs. E2 + DPN + water; *P* < 0.05), and was reversed with 3-MA treatment (E2 + DPN + 3-MA vs. E2 + DPN; E2 + DPN + 3-MA vs. E2 + DPN + water; *P* < 0.05). There were no significant differences for the expression of mTOR both at mRNA and protein levels among groups. However, the protein expression of p-mTOR was downregulated with DPN treatment (E2 vs. E2 + DPN, *P* < 0.01; E2 vs. E2 + DPN + water; *P* < 0.05), and was reversed with 3-MA treatment (E2 + DPN + 3-MA vs. E2 + DPN; E2 + DPN + 3-MA vs. E2 + DPN + water; *P* < 0.05). Those findings demonstrated that ERβ induced autophagy by reducing the level of P62 and mTOR.

## Discussion

ERs were reported to be involved in the nosogenesis and tumor progression of many cancer, such as prostatic cancer, breast carcinoma, ovarian cancer, and others [16]. ERβ was found to exert tumor-suppressive effects in cancers, suggesting the therapeutic target role of ERβ [17, 18]. For example, a study suggested that Erβ served as a tumor suppressor in prostate cancer by inhibiting the expression and activity of androgen receptor [18]. In addition, ERβ agonists had been selected to prevent and treat in several cancers [19–21]. Wei et al. reported that the ERβ-mediated cyclin D1 degradation could repress the proliferation of colon cancer cells by autophagy [22]. In addition, a study had revealed that ERβ overexpression served a neuroprotective role by the interaction with ATG7 and the activation of autophagy—lysosomal [23]. In the current study, we found that the ERβ agonist (DPN) inhibited the osteosarcoma cell viability and mediated the osteosarcoma cell death by inducing apoptosis and autophagy. This was consistent with the above views.

Autophagy was considered as a type II programmed cell death associated with the regulation of intracellular homeostasis [24]. Autophagy referred to a lysosomal degradation process starting at the formation of autophagosomes, and it was involved in multiple physiological processes, such as cell survival, differentiation and death, and pathological processes [24, 25]. Changes of autophagy activity are associated with the development and progression of malignant tumors, and the role of autophagy in anti-tumor therapy is still debatable [25]. LC3 was microtubule-associated protein which was widely present in tissues and cells of mammal [26]. LC3 ubiquitin-like modifications were implicated in the formation of autophagosomes [26]. LC3-I was converted to LC3-II which was transposed to the autophagosome membrane during autophagy, and LC3-II had been considered as an autophagosomal marker [27]. This conversion was also observed in our study.

P62, also named sequestosome 1 or ubiquitin-binding protein P62, was the first identified autophagy adaptor [28]. p62 is also a signaling hub by interacting with key signaling proteins; studies on p62 in cell signaling and autophagy would be extremely important for therapeutic exploitation of such an adaptor protein [28]. In addition, it was reported that P62 could be directly bound to LC3 through a specific motif, and contributed to the connection of ubiquitinated proteins to autophagy mechanism, so that they could be degraded in the lysosome [27]. Xu et al. demonstrated Sp1 transcription factor (SP1) could increase the expression of p62 via binding to p62 promoter, and SP1 could decrease autophagy flux by the activation of p62, while SP1 deficiency could increase autophagy flux in gastric cancer [29]. Zhang et al. suggested that DEAD box protein 5 could inhibit tumorigenesis in hepatocellular carcinoma through inducing autophagy by interacting with p62/sequestosome 1 [30]. In our study, the expression of P62 was significantly decreased with the DPN treatment, while the p62 expression was increased when treated with 3-MA, suggesting that ERβ activation did induce autophagy. Those results were consistent with the previous reports above.

As far as we know, mTOR was a crucial factor in the regulation of autophagy and served an important role in mediating the balance among cell growth and autophagy in answer to nutriture and other signals. The deregulation of the mTOR signaling pathway had been reported to be involved in various pathological diseases and tumor progression, including osteosarcoma [31, 32]. In osteosarcoma, activation of mTOR signaling (phosphorylation) accelerated cellular metastasis and presented an unfavorable prognosis through the regulation of downstream targets [32]. Kim et al revealed that deoxypodophyllotoxin triggered autophagy in osteosarcoma U2-OS cells which was cytoprotective via repressing phosphoinositide-3-kinase (PI3K) /AKT/mTOR pathway [33]. In addition, it had reported that autophagy and apoptosis were triggered in osteosarcoma MG-63 cells with honokiol treatment, which was associated with increasing the expression of LC3II while decreasing PI3K, phosphorylation-Akt, and p-mTOR expression levels [34]. Similarly, p-mTOR expression was significantly reduced with DPN treatment while increased with 3-MA treatment in our study. From the above, we concluded that ERβ repressed osteosarcoma progression through triggering autophagy which was associated with the expression of P62 and p-mTOR. However, the specific regulatory mechanism had not been clearly explored; for instance, the downstream effectors were need to be investigated.

## Conclusion

In a word, ERβ inhibited cell viability and mediated cell death by inducing apoptosis and autophagy in osteosarcoma. ERβ-induced autophagy in osteosarcoma was associated with downregulating the expression of P62 and p-mTOR. The current study revealed that ERβ might be a promising target for the study and the treatment of osteosarcoma.

## Data Availability

The datasets used and/or analyzed during the current study are available from the corresponding author on reasonable request.

## Abbreviations

3-MA: 3-Methyladenine
CCK-8: Cell counting kit-8
ECL: Enhanced chemiluminescence
ER: Estrogen receptors
ERβ: Estrogen receptor beta
MOI: Multiplicity of infection
mTOR: Mammalian target of rapamycin
PI3K: Phosphoinositide-3-kinase
p-mTOR: phosphorylated-mTOR
RT-qPCR: Reverse transcription quantitative polymerase chain reaction
SD: Standard deviation
